# The Impact of Resveratrol-Enriched Bread on Cardiac Remodeling in a Preclinical Model of Diabetes

**DOI:** 10.3390/antiox12051066

**Published:** 2023-05-09

**Authors:** Andreia F. R. Silva, Rita Silva-Reis, Rita Ferreira, Paula A. Oliveira, Ana I. Faustino-Rocha, Maria de Lurdes Pinto, Manuel A. Coimbra, Artur M. S. Silva, Susana M. Cardoso

**Affiliations:** 1 LAQV-REQUIMTE, Department of Chemistry, University of Aveiro, 3810-193 Aveiro, Portugal; afrs@ua.pt (A.F.R.S.); reis.rita@ua.pt (R.S.-R.); mac@ua.pt (M.A.C.); artur.silva@ua.pt (A.M.S.S.); 2 Center for the Research and Technology of Agro-Environmental and Biological Sciences (CITAB), Inov4Agro, University of Trás-os-Montes and Alto Douro (UTAD), 5000-801 Vila Real, Portugal; pamo@utad.pt (P.A.O.);; 3 Department of Veterinary Sciences, University of Trás-os-Montes and Alto Douro UTAD, 5000-801 Vila Real, Portugal; 4 Department of Zootechnics, Comprehensive Health Research Center, School of Sciences and Technology, University of Évora, 7004-516 Évora, Portugal

**Keywords:** type 2 diabetes, stilbene, Sprague Dawley rats, functional food, bioactive ingredient, cardioprotective

## Abstract

The World Health Organization aims to stop the rise of diabetes by 2025, and diet is one of the most efficient non-pharmacological strategies used to prevent it. Resveratrol (RSV) is a natural compound with anti-diabetic properties, and incorporating it into bread is a suitable way to make it more accessible to consumers as it can be included as part of their daily diet. This study aimed to evaluate the effect of RSV-enriched bread in preventing early type 2 diabetes cardiomyopathy in vivo. Male Sprague Dawley rats (3 weeks old) were divided into four groups: controls with plain bread (CB) and RSV bread (CBR), and diabetics with plain bread (DB) and RSV bread (DBR). Type 2 diabetes was induced by adding fructose to the drinking water for two weeks followed by an injection of streptozotocin (STZ) (40 mg/kg). Then, plain bread and RSV bread (10 mg RSV/kg body weight) were included in the rats’ diet for four weeks. Cardiac function, anthropometric, and systemic biochemical parameters were monitored, as well as the histology of the heart and molecular markers of regeneration, metabolism, and oxidative stress. Data showed that an RSV bread diet decreased the polydipsia and body weight loss observed in the early stages of the disease. At the cardiac level, an RSV bread diet diminished fibrosis but did not counteract the dysfunction and metabolic changes seen in fructose-fed STZ-injected rats.

## 1. Introduction

Cardiovascular diseases (CVD) are the leading cause of death worldwide, accounting for approximately 32% of all deaths in 2021. This number is expected to continue to rise in the coming years [1]. A sedentary lifestyle, psychosocial stress, and unhealthy diets are the main modifiable risk factors for CVD in the population. These factors, when combined with non-modifiable risk factors such as age, genetic medical history, and other diseases, further increase the incidence of CVD [2,3]. Individuals with type 2 diabetes mellitus are at high risk for CVD, with up to a fourfold risk of stroke and a twofold risk of dying from myocardial infarction. Data from the International Diabetes Federation reveal a concerning rise of 16% in the number of diabetic adults worldwide since 2019, with around 90% being type 2 [4]. Diabetes cardiomyopathy is characterized by structural and functional changes in the heart, accelerating myocardial dysfunction. These changes include fibrosis, hypertrophy, reduced diastolic compliance, and cardiomyocyte apoptosis [5]. Given the clinical impact of diabetes as a cardiovascular risk factor, there has been a growing focus on implementing therapeutic strategies, pharmacological and non-pharmacological ones, to prevent or counteract diabetes-related cardiac maladaptive remodeling. 

Diet is one of the most effective non-pharmacological strategies for CVD prevention and management. Consistent observations from various studies support the benefits of dietary habits towards consuming products rich in bioactive compounds, such as fruits and vegetables, olive oil, and whole grains [6,7,8]. In this context, *trans*-resveratrol (RSV), a natural stilbene found in grapevine *Vinis vinifera*, wine, and peanuts [9,10] and approved for consumption as an ingredient at 150 mg of RSV/day maximum [11], is quite interesting. RSV bioactivities comprise decreasing lipid peroxidation, platelet aggregation, vasodilation, and blood pressure. In the context of diabetes, RSV has been shown to improve the serum cholesterol and triglycerides profile, and glucose homeostasis by diminishing hyperglycemia, promoting insulin sensitivity, and protecting the islet β-cells [12,13]. 

In vivo studies have provided evidence of the health benefits of RSV in diabetes prevention. For example, a study conducted on diabetic adult mice revealed that a diet enriched with 100 mg of RSV/kg body weight (BW) per day for a year improved their cardiac function. The underlying protective mechanism seems to involve the activation of the NAD^+^-dependent deacetylase SIRT1 and the overexpression of sarcoplasmic calcium pump ATPase (SERCA), an important player in the regulation of heart contractibility [14]. Similarly, in diabetic Goto-Kakizaki rats, the intragastrical administration of 20 mg of RSV/kg BW for ten weeks improved glucose tolerance and homeostasis via SIRT1 activation coupled with the stimulation of AMP-activated protein kinase (AMPK) [15]. Moreover, diabetic Sprague Dawley rats treated with 1 mg of RSV/kg BW per day for five days in combination with insulin (4 IU/rat/day) showed improved systolic cardiac function and reduced oxidative stress and mortality rate [16]. A pilot study conducted on age-related glucose-intolerant people highlighted that RSV can improve insulin sensibility and meal tolerance [17]. However, the relationship between RSV doses and biological effects does not seem linear [18]. Clinical trials have demonstrated that RSV doses of 250 mg daily for three months, or 10 mg, 1 g, 1.5 g, and 2 g daily for one month, can improve insulin sensibility and glycemic index. Still, no effect was demonstrated in a trial with 500 mg of RSV twice daily for twenty-five weeks with washout periods [19]. 

The incorporation of RSV into processed food products has been proposed as a means to increase its daily consumption due to the low concentration of RSV in natural food products. Liquid food products have been fortified with RSV [20,21,22], and bread has been modified to act as a vehicle for RSV intake, allowing the protection of RSV in the bread-making process [23]. In this way, the consumption of bioactive compounds that are typically scarce in daily dietary habits can increase [24,25]. Thus, this study aims to evaluate the effect of functional bread with RSV in preventing early type-2-diabetes-induced cardiac remodeling through functional, histological, and molecular evaluations of cardiac muscle in a non-genetic rat model. Fructose-fed plus STZ-injected rats were used as a preclinical model of type 2 diabetes since it is cost-effective, easy to develop, and displays the same heart clinical features (e.g., stroke volume (SV) and peak filling rate (PFR) echography parameters, hypertrophy, and fibrosis) observed in humans with prediabetes [26,27,28,29].

## 2. Materials and Methods

### 2.1. Ethical Statement

The University Ethics Committee ORBEA—Órgão Responsável pelo Bem-Estar e Ética Animal—approved the experimental protocol, number 956-e-CITAB-2021. All the experiments performed on the animals were carried out under European and National legislation on the protection of animals used for scientific purposes (European Directive 2010/63/EU and Decree-Law no. 113/2013, respectively). The animal experiment was conducted at the animal house of the University of Trás-os-Montes and Alto Douro (UTAD), Portugal. 

### 2.2. Rodents Food Preparation

Food preparation calculations were done assuming that rats need approximately 60 calories per day, equivalent to 5 to 6 g (0.16 to 0.19 oz) of food per 100 g (3.22 oz) of BW of the rat per day. The bread was prepared according to the base recipe reported [23]: 100 g of white wheat flour, 60 g of water, and 3 g of baker’s yeast, kneading until obtaining a non-stick-to-the-hand dough, left to rest for two hours in a warm and dry place, and baked in an oven preheated at 180 °C. All the bread was freeze-dried and milled to be well preserved until incorporation in rodent food pellets in an amount of 1% by Envigo (Barcelona, Spain). 

### 2.3. Animal Housing and Chemicals

Forty-eight male Sprague Dawley rats who were three weeks old were bought from Envigo (Barcelona, Spain), and they remained for one week for acclimatization. The animals were kept in polycarbonate cages with smooth surfaces and rounded edges (1500U Eurostandard Type IV S, Tecniplast, Buguggiate, Italy), with three to five animals per cage. The bedding for the animals (corncob, Ultragene, Santa Comba Dão, Portugal) was changed every week, and in the case of diabetic animal groups, it was changed daily up to the third week of the experiment. The housing conditions were controlled during all experiments to a temperature of 22 ± 2 °C, humidity 60%, and light/dark cycle 12 h/12 h. 

The food (Safe Diets, Augy, France) and water were ad libitum. Fructose and streptozotocin (STZ) were obtained from BioPortugal- Químico, Farmacêutica, Lda (Porto, Portugal). 

### 2.4. Experimental Design

The animals were randomly divided into four groups, two control and two experimental groups. The control animals were fed plain bread (CB, n = 8) and bread with 10 mg/kg/day of RSV (CBR, n = 8); the fructose-fed and STZ-injected (fructose/STZ) animals were fed plain bread (DB, n = 16) and bread containing 10 mg of RSV/kg/day (DBR, n = 16). The number of animals in fructose/STZ groups was double that of the control groups due to the expected higher mortality rate.

**Figure 1:**
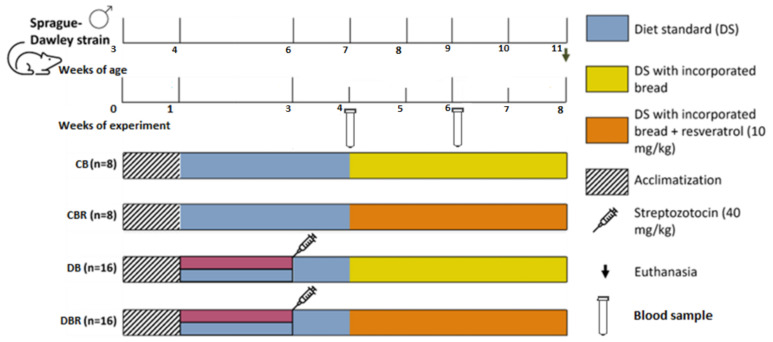
Experiment protocol scheme.

The experiment started with one week of acclimatization, with a standard diet and plain water. After that period, the drinkable water of groups DB and DBR was replaced by 10% fructose water, prepared daily over 14 days to decrease the insulin sensibility, followed by an intraperitoneal STZ injection (40 mg/kg), prepared in a 0.1 M citrate buffer solution (pH 4.3) (week 3 of the experiment). The control groups, CB and CBR, kept drinking plain water and were injected with the vehicle instead of the STZ solution. In week 4 of the experiment, a standard diet enriched with plain bread was introduced in the CB and DB groups, and the CBR and DBR groups had access to a standard diet containing bread with 10 mg of RSV/kg/day. The experiment finished at the end of week 8 (Figure 1). 

Glucose levels were measured in blood samples collected from the rats’ tail veins using a glucometer (GlucoMen^®^ Areo 2K, A. Menarini Diagnostics, Florence, Italy) and blood test strips. Measurements were taken 2 h and 12 h after a meal during two time points: one week after the STZ injection (week 4 of the experiment) and two weeks after introducing a standard diet with bread (week 6), according to Figure 1. Body weight, water, and food intake were also monitored throughout the experiment. As the animals were housed in groups, food and water consumption was determined by measuring the amount of food and water intake in each cage. Animal welfare was evaluated using a humane endpoints table (Supplementary Table S2) based on CCAC guidelines and adapted from previous studies [30,31]. Several parameters were monitored, including posture, hair/tail appearance and grooming, Grimace scale (i.e., eyes and extremities, the position of ears and whiskers, nose/cheeks), walk, skin, mental and hydration status, response to external stimuli, stool appearance, convulsions, and response to mild abdominal palpation. For each parameter, a score from 0 to 3 was assigned to each animal. Rats were observed daily, and animal scores were recorded weekly. The score for each animal was calculated by summing the scores allocated to each parameter. If the animals’ humane endpoints score achieved the critical level, i.e., four or higher, they ought to be re-evaluated and, if necessary, removed from the study and humanely euthanized. Additionally, a score of three in certain parameters, such as body mass and mental condition, also indicated the need for euthanasia.

After an overnight fast of 12 h, the animals were weighed and anesthetized with ketamine/xylazine. Echocardiography was then performed before the animals were euthanized by exsanguination through cardiac puncture. Blood was collected for serum separation by centrifugation and stored at −80 °C for biochemical analysis. A complete necropsy was performed on each animal. The heart was divided into two parts, the apex was immediately processed for histological analysis, and the other portion was stored at −80 °C for biochemical analysis. 

### 2.5. Echocardiographic Study

Immediately before the animals’ sacrifice, a transthoracic echocardiographic study was performed using the Logiq P6 (General Electric Healthcare, Milwaukee, WI, USA) ultrasound device equipped with a 12 MHz linear transducer. For this, the left region of the thoracic wall of the anesthetized animals was shaved using a machine clipper. The animals were placed in left lateral recumbency and the transducer was applied directly to the shaved region. Parasternal long-axis (PLAX), parasternal short-axis (PSAX), four-chamber view, and apical five-chamber view were obtained using B-mode, M-mode, Color Doppler, and Pulsed Doppler. The images were recorded in the ultrasound device and then exported and analyzed using Horos (https://horosproject.org, accessed on 12 November 2021), except for the aortic velocity time integral (AoVTI) measured in the ultrasound device. Measurements were obtained from standard views according to the accepted standards for rats. 

The same researcher analyzed two representative cardiac cycles, and a mean value was calculated for each measurement. The thickness of the intraventricular septum (IVS) and left posterior ventricular wall (LVPW) in systole and diastole were obtained in PLAX B-mode. The left ventricle mass (LV mass) was calculated using the Troy formula, corrected for the body surface area. The aortic root diameter (Ao d) was measured in PLAX M-mode. The aortic velocity time integral (AoVTI) was measured using the ultrasound device in the apical five-chamber view. The heart rate (HR) was measured in PSAX M-mode. The left ventricle filling (E and A wave velocities) was assessed using Pulsed Doppler transmitral flow velocity tracings obtained just above the tip of the mitral leaflets in the four-chamber view. Fractional shortening (FS), stroke volume (SV), cardiac output (CO), and ejection fraction (EF) were obtained by applying formulas. FS and EF were derived from the left ventricle diameter at systole and diastole. SV was derived from Ao d and AoVTI, while CO was derived from SV and HR. 

### 2.6. Biochemical Analysis in Serum Samples

Glucose (Glc) and triglycerides (TG) were assessed in blood-derived serum using Liquick Cor kits (Cormay, Lomianki, Poland) according to the manufacturer’s instructions. Insulin concentrations were measured by a mouse insulin ELISA kit (Invitrogen, Thermofisher, Waltham, MA, USA) according to the company’s instructions. The insulin sensitivity was assessed by calculating the quantitative insulin sensitivity check index (QUICKI), as previously reported [32]. For the determination of the inflammatory parameter C-reactive protein (CRP), and protein carbonylation and nitration, known markers of oxidative stress, slot-blot was performed. In brief, equal volumes of serum samples from animals of each group were slot-blotted under vacuum in a nitrocellulose membrane (Amersham™ Protan™, GE Healthcare Lifesciences) and incubated for 1 h with a primary antibody (rabbit anti-CRP, 1:1000, ab65842, Abcam, Cambridge, UK; mouse anti-nitrotyrosine (nitroTyr), MAB5404, Millipore Sigma, St. Louis, MO, USA; mouse anti-dinitrophenylhidrazone (DNP), 1:1000, MAB2223, Millipore Sigma). Incubation with secondary antibody and signal detection are detailed in Section 2.9.

### 2.7. Histological Analysis

Heart sections (apex side) were fixed in 4% (*v*/*v*) buffered paraformaldehyde, dehydrated through graded ethanol, and included in paraffin blocks. Consecutive sections (3 μm of thickness) of paraffin blocks were cut using a microtome and mounted on silane-coated slides. The slides were dewaxed in xylene, hydrated through graded ethanol, and washed in water. To determine the cardiomyocyte cross-sectional area (CSA) and fibrosis, the deparaffinized sections of cardiac tissue were stained with Sirius Red. 

The digital images of tissue sections were captured using an optical microscope Zeiss Axio Imager Z1 (Carl Zeiss, Jena, Germany) equipped with a CCD monochromatic digital camera (Axiocam HRm, Jena, Germany) at ×200 and Axiovision software (Carl Zeiss, Jena, Germany). Structural changes in the heart tissue were qualitatively evaluated through a visual inspection of the colored photomicrograph images captured at ×200 magnification. The CSA of at least 500 cardiomyocytes per group was determined using ZEN Lite software (ZEN v3.2 (blue edition), Carl Zeiss, Jena, Germany).

### 2.8. Cardiac Muscle Preparation for Biochemical Analysis

Cardiac muscle sections were homogenized in HEPES buffer 10 mM, pH 7.4 with 0.1% Triton X-100 supplemented with protease inhibitor (200 mM PMSF), in the proportion of 50 mg/mL, using a Teflon pestle on motor-driven Potter–Elvehjem glass. The protein content of the cardiac muscle extracts was determined using the DC™ Protein Assay (Bio-Rad, Hercules, CA, USA) and following the manufacturer’s instructions. Bovine serum albumin was used as the protein standard.

### 2.9. Immunoblotting

Briefly, 30 μg of protein was diluted (1:2) in loading buffer (4% SDS (*w*/*v*); 125 mM of Tris, pH 6.8; 15% glycerol (*v*/*v*); 20% β-mercaptoethanol (*v*/*v*); 0.1% bromophenol blue) and incubated at 100 °C for 5 min. Loaded samples were electrophoresed on 12.5% SDS-PAGE at 200 volts, as previously described [33]. Gels were blotted onto a nitrocellulose membrane (Amersham™ Protan™, GE Healthcare Lifesciences, Milwaukee, WI, USA) at 200 mA for 2 h. The non-specific binding was blocked with 5% (*w*/*v*) non-fat dry milk in TBS-T (100 mM Tris, 1.5 mM NaCl, 0.5% Tween 20). Then, membranes were incubated with primary antibody to detect ATP synthase subunit beta (mouse anti-ATPβ, 1:1000, ab14730, Abcam), phosphofructokinase, muscle isoform (rabbit anti-PFKM, 1:1000, ab154804, Abcam), CAAT enhancer-binding protein beta (rabbit anti-CEBPβ, 1:1000, ab32358, Abcam), Cbp/P300-Interacting Transactivator 4 (rabbit anti-CITED4, 1:1000, mbs833529, MyBioSource, San Diego, CA, USA), connexin 43 (rabbit anti-Cx43, 1:1000, ab11370, Abcam), sirtuin 3 (rabbit anti-SIRT3, 1:1000, 26275, Millipore Sigma), glucose transporter type 4 (mouse anti-GLUT4, 1:1000, ab48547, Abcam), manganese superoxide dismutase (rabbit anti-MnSOD, 1:1000, ab13533, Abcam) or Electron Transfer Flavoprotein Dehydrogenase (rabbit anti-ETFDH, 1:1000, ab91505, Abcam). For protein carbonylation analysis, the samples were derivatized with 2,4-dinitrophenylhydrazine (DNPH) before electrophoresis, as described [34], and incubated with an anti-DNP antibody (mouse, 1:1000, MAB2223, Millipore Sigma). For the analysis of protein nitration in cardiac extracts, 10 μg of protein was slot-blotted into a nitrocellulose membrane (Amersham™ Protan™, GE Healthcare Lifesciences) under vacuum and incubated with anti-nitroTyr antibody (mouse, 1:1000, MAB5404, Millipore Sigma). Membranes were incubated for 1 h at room temperature or overnight at 4 °C. Then, membranes were washed 3 times for 10 min with TBS-T and incubated with secondary Horseradish Peroxidase (HRP)-conjugated anti-mouse or anti-rabbit antibodies (GE Healthcare Life Sciences, Milwaukee, WI, USA; diluted 1:1000 in 5% (*w*/*v*) non-fat dry milk in TBS-T) for 2 h at room temperature. Immunoreactive bands were detected by chemiluminescence (ECL; ClarityTM Western ECL Substrate #170-5060, Bio-Rad, Hercules, CA, USA) according to the manufacturer’s instructions. Images were obtained in a ChemiDoc™ Imaging System (Bio-Rad, Hercules, CA, USA) and analyzed with Image Lab (version 6.0, Bio-Rad, Hercules, CA, USA). The optical density (OD) values were expressed in arbitrary units. Protein loading was controlled by Ponceau S staining.

### 2.10. Citrate Synthase Activity

Citrate synthase (CS) activity was determined in cardiac muscle extracts by monitoring the reduction of 5,5′-dithiobis(2-nitrobenzoic acid) (DTNB) at 412 nm as described in [35]. In brief, DTNB reacted with coenzyme A (thiol group) released by the reaction of acetyl-CoA with oxaloacetate. The reaction was followed spectrophotometrically at 412 nm (molar extinction coefficient of 13.6 mM^−1^·cm^−1^). The results are presented in nmol min^−1^ mg^−1^ protein. 

### 2.11. Statistical Analysis

Values are presented as the mean ± standard deviation for all variables. The Kolmogorov–Smirnov test was performed to evaluate the normality of the data. One-way analysis of variance (ANOVA) followed by Tukey’s multiple comparisons post hoc test was used. Dunn’s multiple comparisons were performed when data did not pass the normality test. Results were considered significantly different when *p* < 0.05, and it was assumed a tendency for *p* < 0.1. GraphPad Prism (version 9.0) was the software used.

## 3. Results

### 3.1. Effect of Functional Bread with RSV and Fructose Feed/STZ Injection on Anthropometric and Welfare Parameters

During the protocol, one animal from the fructose/STZ group fed plain bread died. The mortality rate was 0% for other groups. Body temperature, water intake, food intake, and body weight were monitored throughout the protocol to check on the health status of the animals (Figure S1, supplementary data). A normal body temperature was maintained throughout the experiment. Fructose/STZ animals displayed an increase in food and water intake starting from the third week, with water intake increasing eightfold and food intake increasing onefold. In addition, these animals had excessive urine excretion, supporting the development of diabetes in fructose/STZ rats. 

A significantly lower body weight of around 23% was also noticed in these animals (*p* < 0.05 vs. control groups, Table 1). The gastrocnemius mass was 40% and 30% lower in DB and DBR, respectively, compared with the controls (*p* < 0.05 DB and DBR vs. controls). Fructose/STZ rats showed no signs of impaired growth, given by no changes in the tibia length, but they presented limited weight gain, reflected by the lower body weight/tibia length ratio (Table 1), which significantly increased with an RSV bread diet (*p* < 0.05 DB vs. DBR). Regarding heart weight and the heart weight/tibia length ratio, these parameters were significantly lower in fructose/STZ animals (*p* < 0.05 vs. control groups).

A tendency for a higher welfare score was observed in fructose/STZ rats fed plain bread compared with control rats also fed plain bread (*p* = 0.08 for DB vs. CB; Table 1). Diet with RSV bread promoted a decrease in the welfare score in fructose/STZ rats (0.5 in DBR vs. 1.13 in DB, Table 1). According to the adapted Canadian Council on Animal Care—CCAC guidelines, the welfare scores in this study were below the critical animals’ welfare score of three [30,31]. Thus, the experimental protocol was deemed harmless, and by incorporating RSV into the bread diet, the animals’ welfare score trend was improved. 

### 3.2. Systemic Adaptations to Fructose-Feeding plus Streptozotocin Administration and Functional Bread with RSV 

Hyperglycemia development in fructose/STZ rats was confirmed through the monitoring of glucose (Glc) blood levels at fasting and feeding conditions at weeks 4 and 6 of the experiment (Figure S2a,b, supplementary data). Prediabetes is diagnosed in rats when fasting Glc levels are between 200 and 250 mg/dL, and diabetes when equal to or higher than 250 mg/dL [36]. According to Figure S2a, at week 4 and week 6, the feeding glycemia was higher than 200 mg/dL in fructose/STZ rats and close to 141 mg/dL in the control groups. However, fasting Glc levels were still below 200 mg/mL at week 6 in fructose/STZ groups (Figure S2b).

**Figure 2:**
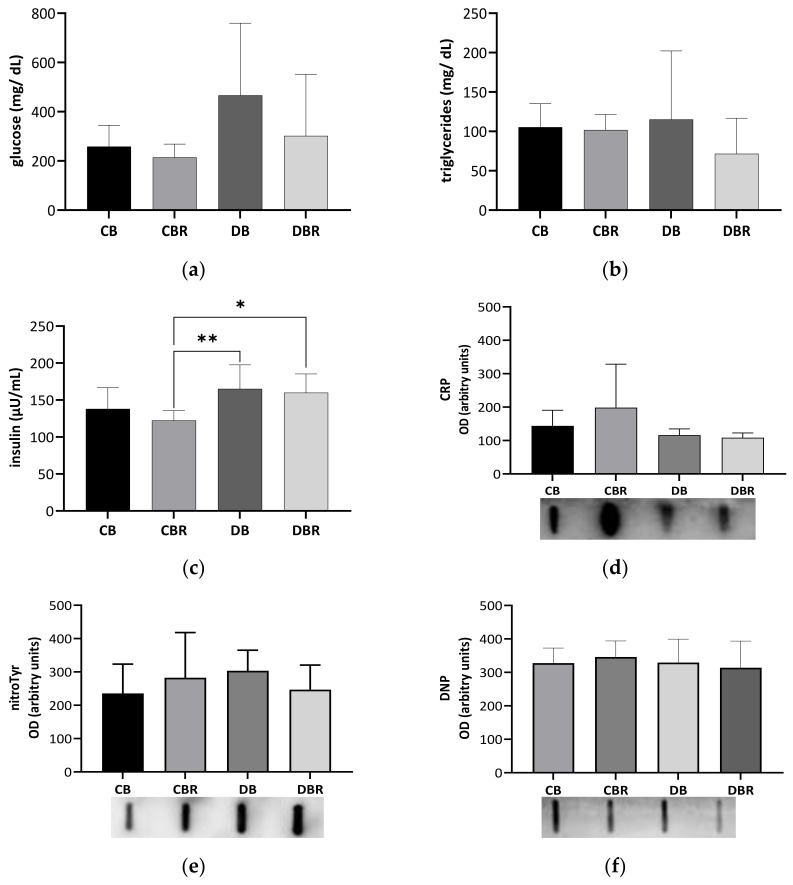
Circulating levels of glucose (**a**), triglycerides (**b**), and insulin (**c**), C-reactive protein (CRP) **(d**), 3-nitrotyrosine (nitroTyr), and (**e**) dinitrophenol (DNP) (**f**) assessed in blood-derived serum samples collected at necropsy. Values represent mean ± standard deviation in each group (* *p* < 0.05, ** *p* < 0.01). CB is the control group fed plain bread (n = 6), and CBR is the group fed bread with 10 mg/kg of RSV a day (n = 6); DB is the fructose/STZ group fed plain bread (n = 6), and DBR is the one fed bread containing 10 mg/kg of RSV a day (n = 6).

Circulating Glc, triglycerides (TG), and insulin levels were determined in blood samples collected at necropsy (Figure 2a–c). Glc levels tended to be twofold higher in fructose/STZ rats compared with controls fed RSV bread (*p* = 0.08 for DB vs. CBR), confirming fasting hyperglycemia at the end of the protocol (Figure 2a). The high variability of the circulating Glc levels noticed among fructose/STZ rats seems to be indicative of the acute glucose fluctuations seen in the initial stages of diabetes, also known as dysglycemia [37]. Moreover, TG serum levels showed no significant differences between groups, with all animals presenting similar values that were within the reference range for Sprague Dawley rats (20.35–87.61 mg/dL) [38]. On the other hand, fructose/STZ animals fed RSV bread exhibited higher insulin levels compared to those fed plain bread (*p* < 0.05 DBR vs. CBR and DB vs. CBR), which may indicate β-cell hyperfunction to overcome insulin resistance [39]. The assessment of insulin sensitivity through QUICKI revealed values of 0.223 ± 0.008 and 0.226 ± 0.005 for the control groups fed plain and RSV bread, respectively, and 0.210 ± 0.016 and 0.217 ± 0.014 for fructose/STZ animals, respectively. Statistical differences were observed between DB and CBR (*p* < 0.05), as also noticed in insulin secretion values, suggesting less insulin sensibility in fructose/STZ animals fed plain bread.

To investigate whether fructose/STZ-induced diabetes is linked with inflammation, we assessed the levels of CRP in the blood-derived serum. However, no significant changes were observed in the levels of this acute-phase protein (*p* > 0.05; Figure 2d), suggesting that there is no systemic inflammation at this stage of the disease. Furthermore, no alterations were seen in the levels of protein carbonylation and nitration (*p* > 0.05; Figure 2e,f), which are known markers of oxidative stress. These findings suggest that at this stage of the disease, fructose/STZ-induced diabetes is not primarily driven by systemic inflammation and oxidative stress.

### 3.3. The Influence of Fructose-Feeding plus Streptozotocin Administration and Functional Bread with RSV on Cardiac Function and Morphometry 

The cardiac structure and function parameters of the four groups determined by echocardiographic evaluation are presented in Table 2. The intraventricular septum thickness (IVS) and left ventricular posterior wall thickness (LVPW) similarities between DB and CB groups suggest that fructose plus STZ did not interfere with these parameters but rather increased the left ventricular ejection time (LV ET) (*p* < 0.05 DB and DBR vs. CB), corroborating the reduced cardiac output (CO) and heart rate (HR) seen in these animals (*p* < 0.05 DB vs. CB). An RSV bread diet increased the LVPW and IVS values in fructose/STZ groups (*p* < 0.05 DBR vs. CBR and *p* < 0.05 for DB vs. DBR), suggesting some beneficial effect of RSV on fructose/STZ-associated cardiac adaptation. This contributes to the higher left ventricle mass (LV mass), with no significant changes in the left ventricle ejection time (LV ET). The shortening of left ventricle parameters (FS) was also higher in control and fructose/STZ groups fed RSV bread (*p* < 0.05 CBR and DBR vs. CB). The diastolic function indicator E/A was particularly higher in the control fed RSV bread group (*p* < 0.05 CB and DB vs. CBR).

**Figure 3:**
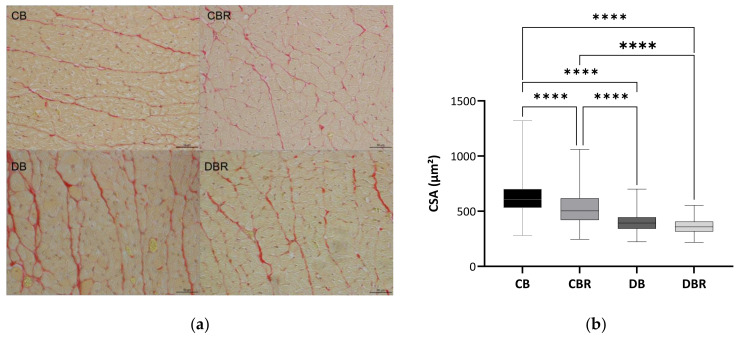

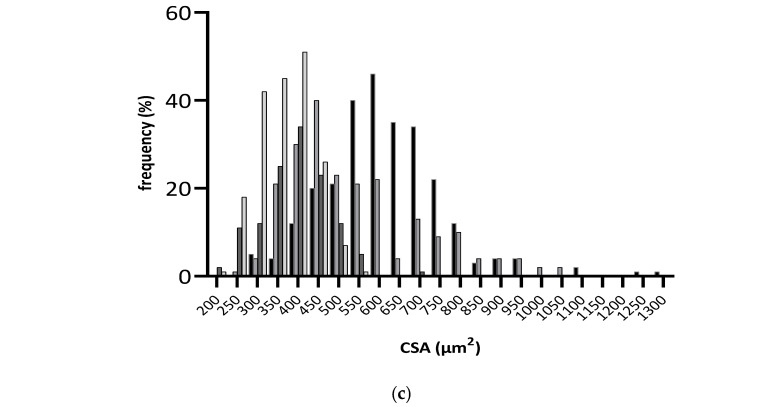
Histological assessment of heart tissue sections stained with Sirius Red, where red areas represent collagen accumulation (**a**); boxplot of the cross-sectional areas (CSA) of cardiomyocytes (**b**): middle line represents the mean and whiskers the minimum and maximum area (**** *p* < 0.0001); and frequency distribution of CSA of cardiomyocytes (**c**). CB is the control group fed plain bread (n = 5), and CBR is the group fed bread with 10 mg/kg of RSV a day (n = 6); DB is the fructose/STZ group fed plain bread (n = 5), and DBR is the one fed bread containing 10 mg/kg of RSV a day (n = 5).

At the cellular level, differences in cardiomyocytes CSA were observed between groups (Figure 3b,c), with significantly lower values observed in fructose/STZ rats. Moreover, a higher frequency of smaller cardiomyocytes was noticed in these animals, between 350 and 450 µm^2^ (Figure 3c). These results do not support HW/BW ratios, which were higher in DB and DBR groups (Table 1). Nevertheless, the qualitative analysis of tissue sections stained with Sirius Red revealed an increase in the staining intensity of the interstitial space in cardiac muscle sections of DB rats and, in a lesser extension, in the DBR group (Figure 3a). Thus, the increased accumulation of densely packed collagen fibers in interstitial space, a sign of cardiac dysfunction, seems to occur in fructose/STZ rats.

### 3.4. The Effect of Fructose-Feeding plus Streptozotocin Administration and Functional Bread with RSV on Molecular Markers of Cardiac Functionality and Regeneration

**Figure 4:**
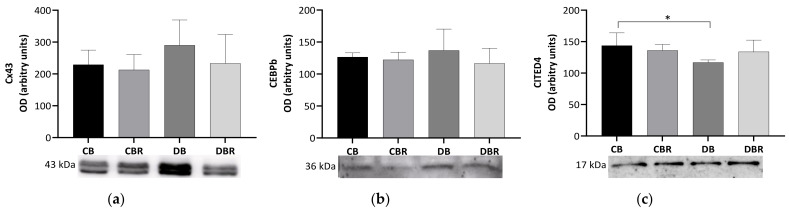
Effects of RSV bread diet on cardiac function and regeneration: connexin 43 (Cx43) (**a**), Cbp/p300-interacting transactivator 1 (CITED) (**b**) and CCAAT/enhancer-binding protein beta-2 isoform (CEBPβ) (**c**). Values represent mean ± standard deviation in each group (* *p* < 0.05). CB is the control group fed plain bread (n = 6), and CBR is the group fed bread with 10 mg/kg of RSV a day (n = 6); DB is the fructose/STZ group fed plain bread (n = 6), and DBR is the one fed bread containing 10 mg/kg of RSV a day (n = 6).

The content of connexin-43 (Cx43), a gap junction channel protein that regulates the passage of electrical impulses and small metabolites [40], was assessed in whole cardiac tissue extracts. No significant differences in the Cx43 content were observed between groups (Figure 4), suggesting no alterations in the regulation of cardiomyocyte electric activity.

We also examined levels of CCAAT/enhancer-binding protein beta-2 isoform (CEBPβ), a DNA-binding transcription factor linked to cell proliferation, differentiation, and non-pathologic cardiac hypertrophy [41,42]. However, no differences were observed among the groups in the levels of this protein. Another protein of interest is CITED4, named Cbp/p300-interacting transactivator with ED-rich carboxy-terminal domain 4, which plays a role in the growth and proliferation of cardiomyocytes [43] and is negatively regulated by CEBPβ [41]. Significantly lower levels of CITED4 were seen in the cardiac muscle from fructose/STZ rats fed plain bread (*p* < 0.05 DB vs. CB) but not in rats fed RSV bread.

### 3.5. The Influence of Fructose-Feeding plus Streptozotocin Administration and Functional Bread with RSV on Cardiac Metabolism and Oxidative Stress

**Figure 5:**
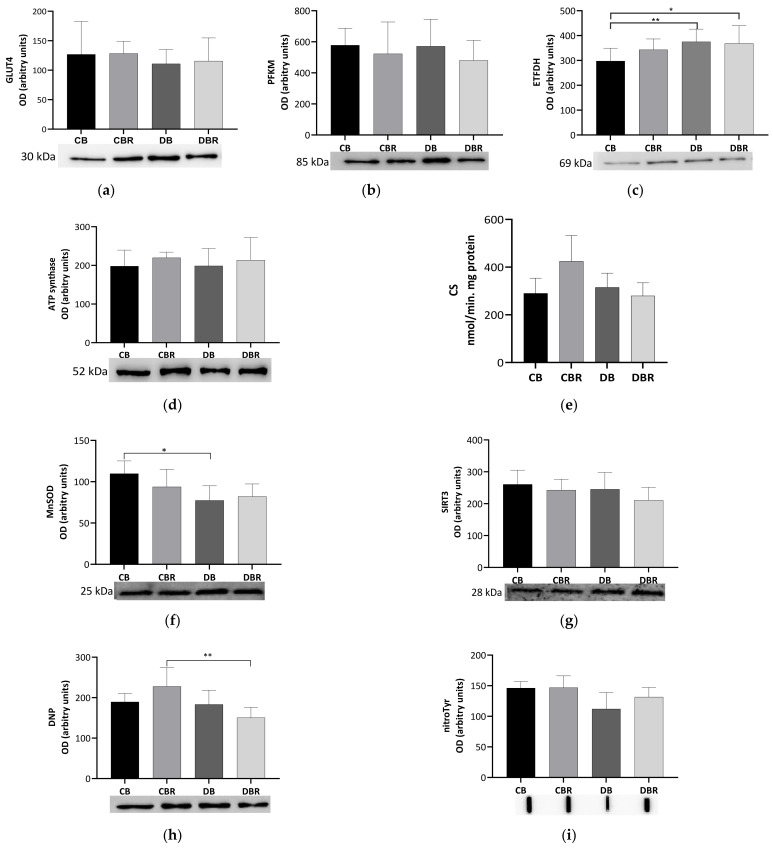
Effects of RSV bread diet on the expression of metabolism biomarkers of cardiac muscle: insulin facilitated glucose transporter member 4 (GLUT4) (**a**), ATP-dependent 6-phosphofructokinase of muscle (PFKM) (**b**), electron transfer flavoprotein–ubiquinone oxidoreductase mitochondrial (ETFDH) (**c**), ATP synthase beta (ATPsβ) (**d**), and citrate synthase (CS) activity (**e**), and oxidative stress biomarkers: manganese superoxide dismutase mitochondrial (MnSOD) (**f**), NAD-dependent protein deacetylase sirtuin-3 (SIRT3) (**g**), dinitrophenol (DNP) for carbonyl groups detection (**h**), and 3-nitrotyrosine (nitroTyr) (**i**). Values represent mean ± standard deviation (* *p* < 0.05, ** *p* < 0.01). CB is the control group fed plain bread (n = 6), and CBR is the group fed bread with 10 mg/kg of RSV a day (n = 6); DB is the fructose/STZ group fed plain bread (n = 6), and DBR is the one fed bread containing 10 mg/kg of RSV a day (n = 6).

To evaluate the metabolic adaptations of cardiac muscle to fructose-feeding plus STZ administration and diet with functional bread with RSV, markers of oxidative and glycolytic metabolism were measured. No differences in the levels of the glycolytic rate-limiting enzyme PFKM were noticed among groups, suggesting no alterations in the reliance on glucose for energetic purposes. A similar profile was noticed for the content of GLUT4, the predominant isoform, and insulin-dependent cardiac glucose transporter [44] (Figure 5a,b).

Meanwhile, significantly higher levels of ETFDH, an enzyme that makes the connection between fatty acid oxidation (FAO) and oxidative phosphorylation (OXPHOS) [45], were noticed in fructose/STZ rats (*p* < 0.05 DB vs. CB and DBR vs. CB; Figure 5c). These results illustrate that the hearts of fructose/STZ rats have a higher energy reliance on FAO for energy production. However, no alterations in the content of ATP synthase subunit beta from OXPHOS complex V were observed (Figure 5d). Moreover, no differences in CS activity, a rough marker of mitochondrial density, were verified in the heart of fructose/STZ rats compared to the controls. Still, a tendency to decrease CS activity was noticed in fructose/STZ animals fed RSV bread compared to their respective controls (*p* = 0.08 DBR vs. CBR, Figure 5e).

The content of the SIRT3, a deacetylase that modulates the activity of metabolic enzymes as OXPHOS complexes [46], was assessed and no differences were seen between groups (Figure 5g). MnSOD, a mitochondrial antioxidant enzyme and a target of SIRT3, was analyzed and significantly lower levels were noticed in fructose/STZ rats compared to the controls (*p* < 0.05 CB vs. DB), except for those fed RSV bread (Figure 5f). Moreover, no significant differences in the levels of carbonylated proteins, a marker of oxidative stress, were observed in fructose/STZ animals. Curiously, a significant decrease in protein carbonylation was noticed when these animals were fed RSV bread compared to their controls (*p* < 0.05 DBR vs. CBR, Figure 5h). Protein nitration was also evaluated. This protein modification results from the reaction of tyrosine residues with peroxynitrite, which is formed in the presence of superoxide anion and nitric oxide [47]. No changes in the protein nitration content were observed between groups (Figure 5i). 

## 4. Discussion

### 4.1. Effect of Resveratrol-Rich Diet on Anthropometric and Systemic Parameters

This study aimed to evaluate the effect of RSV-enriched bread on preventing type-2-diabetes-related cardiomyopathy in a preclinical model. The non-genetic fructose/STZ rat model was chosen because it mimics the clinical pathogenesis seen in humans, particularly insulin resistance and partial pancreatic β-cell dysfunction. In fact, the combination of 10% fructose with a 40 mg/kg dose of STZ, which was used in this study, has been previously shown to induce damage to pancreatic β-cells without promoting their death nine weeks after STZ administration [27]. Since our study evaluated the effects of fructose/STZ five weeks after STZ administration, it is likely that the damage to islet cells was still relatively mild at this point. This may have resulted in higher biological variation in insulin secretion and glucose serum levels, which is consistent with what has been reported in humans with prediabetes [48]. Although no significant differences were seen in the circulating glucose levels in fructose/STZ rats compared to control ones (Figure 2a), 75% of animals from DB groups presented glucose levels higher than 250 mg/dL. Diet with RSV changed this trend, with only 25% of the rats from the DBR group evidencing glucose levels higher than 250 mg/dL. 

At this stage of the disease, fructose/STZ rats also presented polydipsia, polyuria, polyphagia, and body weight loss, further supporting the development of diabetes. Hyperglycemia is known to cause excessive thirst as the body attempts to eliminate excess glucose through increased urine production [39]. In our study, a tendency for RSV to decrease polydipsia was observed in fructose/STZ animals (Figure S1 of supplementary data), possibly by regulating Glc levels [49]. Body weight loss may be a consequence of the reduced uptake of glucose by insulin-responsive tissues such as skeletal muscle and adipose tissue [39]. Indeed, the weight of gastrocnemius decreased 40% in DB and 30% in DBR animals relative to the controls (Table 1). The lesser body weight loss in fructose/STZ animals fed an RSV bread diet highlights the effect of RSV on attenuating insulin resistance, as shown by the higher QUICKI value in the DBR group compared to the DB one. Indeed, these animals kept or even increased the capacity to produce insulin [50], possibly to overcome insulin resistance, which characterizes the early stages of type 2 diabetes [50,51]. Previous studies reported an RSV normalizing effect on glycemia with lower doses of RSV (0.1, 1, and 5 mg of RSV/kg) and less time, and in animals with a more advanced state of hyperglycemia [16,52]. 

In this study, the controls and fructose/STZ animals exhibited normal triglycerides (TG) values, suggesting no changes in lipid metabolism, contrasting with other type 2 diabetes preclinical models that showed high TG levels [53,54,55]. However, none of these studies evaluated the early stages of diabetes. Additionally, we did not observe any signs of systemic inflammation or oxidative stress in the fructose/STZ groups, potentially supporting the early stages of type 2 diabetes.

### 4.2. Effect of Resveratrol-Rich Diet on Heart Remodeling

Diabetes-associated morbidity and mortality are mostly due to complications that may cause debilitating conditions, such as heart failure. Cardiac structural changes, such as left ventricle hypertrophy, are commonly found in type 2 diabetes patients, even in the early stages of the disease [56]. Left ventricle hypertrophy results from the increased thickness of the left ventricular posterior wall (LVPW) and intraventricular septum (IVS), contributing to higher left ventricular mass (LV mass) [56,57,58]. In this study, fructose/STZ animals did not present echography signs of left ventricle hypertrophy. Other parameters are typically lower in diabetes, such as CO [59], Ao _d_ [60], and EF [61,62], also realized in this study, except for Ao _d_. This may be due to the fact that the fructose/STZ animals were in the prediabetic stage. Moreover, the decrease in CO in fructose/STZ rats when compared with control animals may be a consequence of decreased HR in these animals. The lower HR may be linked to a defect in the autonomic regulation of the nervous system, that continuously regulates heart rate, blood pressure, and other activities that maintain homeostasis without conscious effort [63]. These data suggest that the heart of fructose/STZ animals was hyperdynamic, which was previously reported in diabetic patients [64]. Nevertheless, fructose/STZ animals treated with RSV increased EF and showed higher IVS and LVPW, reflected by the higher FS, but without the impairment of the diastolic function commonly associated with type 2 diabetes [65], suggesting that RSV promotes modest cardiac remodeling.

Heart dysfunction has been associated with fibrosis, being usually found in subjects with advanced type 2 diabetes. Hyperglycemia and insulin resistance have been suggested to trigger this fibrotic response [66]. In fact, fibrosis was seen in the heart of fructose/STZ animals, which was related to increased HW/BW despite decreased cardiomyocytes CSA. However, an RSV bread diet tended to diminish this occurrence. In another study with diabetic Sprague Dawley rats fed a high-fat diet and given 10 mg/kg of RSV daily for eight weeks also decreased fibrosis and left ventricle hypertrophy [67]. These observations were concordant with the capacity of RSV to inhibit myocardial fibrosis by inhibiting collagen deposition [68] and by suppressing ROS production [69], precluding improved cardiac functionality. 

The increased fibrosis seen in the heart of fructose/STZ rats was associated with decreased CITED4 levels (Figure 4). Together with CEBPβ, CITED4 regulates cardiomyocyte growth and proliferation [41,43]. Given the protective role of CITED4 against chronic pathological stresses [70], such as hyperglycemia and insulin resistance, lower levels of this protein may contribute to increased fibrosis, HW/BW, and decreased cardiac output. No other molecular signs of maladaptive cardiac remodeling were seen. Rats fed RSV-enriched bread presented values of CITED4 similar to the ones of the control groups, possibly explaining the histological signs of lower collagen deposition, and improved diastolic function.

A similar content of Cx43 was observed among groups, suggesting no alterations in the regulation of the electric activity of cardiac tissue induced by fructose/STZ treatment. The increased expression of this gap junction protein was reported in spontaneously diabetic Goto-Kakizaki rats, when compared with the non-diabetic Wistar-Clear rats [71]. In type 1 diabetic Sprague Dawley rats, Cx43 levels also increased 3 days after the establishment of diabetes, remaining elevated over 35 days of study [72]. Thus, our results possibly reflect an earlier stage of hyperglycemia-induced cardiac maladaptive remodeling. To better explore such remodeling, the main metabolic pathways that support the energetic needs of this organ were assessed. 

In healthy conditions, most of the ATP needed for cardiac muscle contraction and other cellular processes is obtained from FAO (around 60%) and the remaining from glycolysis, which feeds OXPHOS with reducing equivalents. In the heart of fructose/STZ rats, no alterations in the expression of glycolytic PFKM were seen, neither in the content of GLUT4, suggesting no changes in the uptake and oxidation of glucose. However, a significantly higher content of ETFDH was seen in these animals, supporting a higher reliance on FAO for energetic purposes. These results are in accordance with the metabolic adaptation reported for the diabetic heart [73]. The cardiac muscle is highly dependent on GLUT4 for glucose uptake. Thus, low levels of GLUT4 may be expected in cases of insulin resistance, unlike that observed among the groups. Nevertheless, whole tissue levels were assessed, not reflecting the transporter available at the cell membrane for glucose uptake. In fact, a malfunction in GLUT4 translocation rather than a decline in the overall GLUT4 expression was previously reported in the diabetic heart [74]. 

FAO and OXPHOS are metabolic pathways harbored in the mitochondria. Mitochondrial dynamics play a key role in cardiac insulin resistance to support energy production and cell survival [75]. However, no alterations in the mitochondrial density were seen in fructose/STZ rats, as seen by the similar CS activities among groups. The content of ATP synthase subunit beta supports these results. Hence, each mitochondrion must increase its FAO efficiency. Still, the long-term FAO demands may increase reactive oxygen species (ROS) generation, promoting oxidative stress [76]. The defense capacity of cardiac cells against oxidative stress was accessed through the mitochondrial antioxidant enzyme MnSOD, of which the activity was reported to be downregulated by type 2 diabetes [77]. In the present study, fructose/STZ rats had a lower cardiac MnSOD content. This enzyme and others, such as OXPHOS complexes located at mitochondria, are under the control of SIRT3 for their activity regulation through deacetylation [46,78]. RSV was reported to activate SIRT3, thus improving ATP production in the diabetic heart [78,79]. However, there were no significant differences in the SIRT3 content among the experimental groups, and there were no signs of increased ROS production given by protein carbonylation and nitration. These findings are in contrast with other studies that observed an increase in oxidative stress biomarkers and a decrease in antioxidant enzymes in high-fat high-carbohydrates diet prediabetic-induced Sprague Dawley rats [80]. Overall, our data highlight a higher reliance of fructose/STZ rats on FAO to support the energetic needs of the heart, despite no apparent alterations in glycolysis and mitochondrial dynamics and increased ROS generation. 

Taken together, our data support the early stages of maladaptive cardiac remodeling induced by hyperglycemia that resulted in increased fibrosis and HW/BW and decreased cardiac output. Notwithstanding, an RSV-enriched diet prevented some of the cardiac alterations observed in fructose/STZ rats, such as the decrease in the MnSOD and CITED4 expression, prevented extracellular collagen accumulation, increasing IVS, LVPW, FS, and EF cardiac parameters, and improved the animals’ overall welfare by diminishing BW loss and polydipsia. Even in control animals, RSV exerted beneficial effects on overall cardiac function.

### 4.3. Limitations

The present study has some limitations. The histological analysis of pancreas islets was not performed but would have provided a better understanding of the extent of fructose feed plus STZ injection and the effect of RSV on preventing pancreas damage and, thus, glycemia and insulin homeostasis. Nevertheless, this study intended to focus on cardiac remodeling changes and, therefore, general systemic parameters were evaluated, and our focus was on the functional, morphological, and biochemical analysis of the heart. While some indicators of metabolism and oxidative stress were evaluated, the determination of more parameters, particularly regulatory factors, would have provided a more comprehensive analysis of the impact of type 2 diabetes on the early adaptations of cardiac muscle. Finally, a time course study would be relevant to evaluate the effect of an RSV-enriched diet on the development of more advanced stages of type 2 diabetes and to investigate whether the beneficial effects observed in this study are sustained or increased over time.

## 5. Conclusions

Our study shows, for the first time, the impact of a functional bread with RSV integrated into a rodent diet in preventing the early systemic and cardiac adaptations associated with type 2 diabetes in a preclinical model. The RSV-enriched bread diet showed significant improvements in the welfare of fructose/STZ Sprague Dawley rats, specifically by reducing body weight loss and polydipsia. On the heart, RSV has preventive effects against the development of fibrosis and maladaptive cardiac remodeling. Our findings suggest that RSV bread may serve as a dietary intervention strategy for managing the early stages of type 2 diabetes and its associated complications.

## Figures and Tables

**Table 1:** Anthropometric parameters, mortality rate, and welfare score of experimental groups.

	Experimental Groups			
	CB	CBR	DB	DBR
BW (g)	325.4 ± 41.6 ^ab^	343.3 ± 16.0 ^c^	241.9 ± 49.1 ^ac^	263.1 ± 69.2 ^b^
HW (g)	1.16 ± 0.04	1.28 ± 0.11 ^ab^	0.97 ± 0.17 ^a^	1.06 ± 0.23 ^b^
Gastrocnemius (g)	4.60 ± 0.26 ^ab^	4.58 ± 0.14 ^cd^	2.77 ± 0.91 ^ac^	3.22 ± 1.16 ^bd^
Tibia (cm)	4.15 ± 0.13	4.25 ± 0.17 ^a^	4.02 ± 0.19	3.99 ± 0.25 ^a^
BW/tibia (g/cm)	78.4 ± 9.9 ^a^	80.8 ± 3.8 ^bc^	60.0 ± 11.3 ^ab^	64.1 ± 14.2 ^ac^
HW/BW (mg/g)	3.50 ± 0.20 ^ab^	3.72 ± 0.28	4.05 ± 0.43 ^a^	4.09 ± 0.45 ^b^
HW/tibia (mg/cm)	0.284 ± 0.021 ^a^	0.301 ± 0.028 ^bc^	0.225 ± 0.024 ^ab^	0.254 ± 0.040 ^c^
Welfare score	0.13 ± 0.35	0.13 ± 0.35	1.13 ± 1.13	0.50 ± 0.82
Mortality (%)	0	0	6.25%	0

Values are means ± standard deviation of n animals in each group. The same letters represent significative differences (*p* < 0.05), comparing all groups to each other with the same parameter. BW, body weight; HW, heart weight. CB is the control group fed plain bread (n = 8), and CBR is the group fed bread with 10 mg/kg of RSV a day (n = 8); DB is the fructose/STZ group fed plain bread (n = 15), and DBR is the one fed bread containing 10 mg/kg of RSV a day (n = 16).

**Table 2:** Echocardiogram parameters of animal experiment.

	Groups of Treatment			
	CB	CBR	DB	DBR
IVS _d_ (mm)	1.12 ± 0.18 ^a^	1.21 ± 0.20 ^b^	0.968 ± 0.172 ^b^	1.40 ± 0.26 ^ab^
LVPW _d_ (mm)	1.11 ± 0.18 ^a^	1.29 ± 0.29 ^b^	0.994 ± 0.159 ^bc^	1.51 ± 0.34 ^ac^
IVS _s_ (mm)	1.17 ± 0.23 ^a^	1.17 ± 0.18 ^b^	0.956 ± 0.165 ^c^	1.47 ± 0.24 ^abc^
LVPW _s_ (mm)	1.21 ± 0.19 ^a^	1.21 ± 0.24 ^b^	0.925 ± 0.212 ^ab^	1.65 ± 0.38 ^ab^
LV mass (mm)	0.498 ± 0.104 ^a^	0.604 ± 0.130	0.475 ± 0.121 ^b^	0.723 ± 0.250 ^ab^
LV ET (s)	82.9 ± 8.37 ^ab^	93.9 ± 18.3 ^c^	125.8 ± 18.5 ^ac^	108.9 ± 20.0 ^b^
Ao _d_ (cm)	0.283 ± 0.042	0.299 ± 0.029	0.307 ± 0.027	0.283 ± 0.046
Ao VTI (cm)	3.94 ± 0.86 ^a^	5.23 ± 1.20 ^ab^	4.30 ± 0.82	3.81 ± 1.21 ^b^
HR (bpm)	321.3 ± 25.8 ^ab^	272.5 ± 37.9 ^c^	190.0 ± 36.7 ^ac^	217.5 ± 67.3 ^b^
E (cm/s)	0.683 ± 0.117	0.812 ± 0.107 ^ab^	0.600 ± 0.190 ^a^	0.575 ± 0.168 ^b^
A (cm/s)	0.427 ± 0.097 ^a^	0.380 ± 0.149	0.385 ± 0.119	0.308 ± 0.067 ^a^
E/A	1.64 ± 0.29 ^a^	2.47 ± 1.01 ^ab^	1.57 ± 0.21 ^b^	1.90 ± 0.49
FS (%)	6.69 ± 3.30 ^ab^	15.4 ± 7.4 ^a^	11.2 ± 4.76	13.4 ± 6.9 ^b^
SV (mL)	1.12 ± 0.30 ^a^	1.56 ± 0.37 ^ab^	1.36 ± 0.22	1.10 ± 0.45 ^b^
CO (mL/min)	357.6 ± 98.1 ^a^	420.6 ± 98.2 ^bc^	241.2 ± 39.9 ^ab^	292.4 ± 125.9 ^c^
EF (%)	20.1 ± 10.1 ^ab^	38.3 ± 14.7 ^b^	27.9 ± 11.3	37.6 ± 17.1 ^a^

Values are means ± standard deviation of animals in each group. The same letters represent significative differences, a, b, and c (*p* < 0.05), comparing the same parameters between each animal group. In each line, d in subscript means diastole, and s in subscript means systole; IVS—intraventricular septum thickness; LVPW—left ventricular posterior wall thickness; LV mass—left ventricle mass; LV ET—left ventricular ejection time; Ao—aorta; Ao VTI—aortic velocity time integral; HR—heart rate; E—peak early diastolic transmitral flow velocity; A—peak late transmitral flow velocity; FS—fractional shortening of left ventricle; SV—stroke volume; CO—cardiac output; EF—ejection fraction. CB is the control group fed plain bread (n = 8), and CBR is the group fed bread with 10 mg/kg of RSV a day (n = 8); DB is the fructose/STZ group fed plain bread (n = 7), and DBR is the one fed bread containing 10 mg/kg of RSV a day (n = 8).

## Data Availability

The data that support the findings of this study are available on request from the corresponding author.

## Supplementary Materials

The following supporting information can be downloaded at: https://www.mdpi.com/article/10.3390/antiox12051066/s1, Figure S1: Weekly parameters of animal’s housing conditions; Table S1: Animals group and respective treatment of experimental protocol; Figure S2: Circulating glucose levels after feeding and fasting moments of Sprague-Dawley rats in the final of the experiment; Table S2: Humane Endpoints applied to evaluate animal welfare in a fructose/STZ-induced diabetes rat model; Table S3: All echocardiogram parameters of animal experiment; Table S4: Specification of the dilutions and catalog number for each primary antibody.

## References

[1] World Health Organization Cardiovascular Diseases. https://www.who.int/en/news-room/fact-sheets/detail/cardiovascular-diseases-(cvds).

[2] Cannon C.P. (2007). Cardiovascular Disease and Modifiable Cardiometabolic Risk Factors. Clin. Cornerstone.

[3] Francula-Zaninovic S., Nola I.A. (2018). Management of Measurable Variable Cardiovascular Disease’ Risk Factors. Curr. Cardiol. Rev..

[4] International Diabetes Federation Diabetes Now Affects One in 10 Adults Worldwide. https://www.idf.org/news/240:diabetes-now-affects-one-in-10-adults-worldwide.html.

[5] Boudina S., Abel E.D. (2010). Diabetic Cardiomyopathy, Causes and Effects. Rev. Endocr. Metab. Disord..

[6] Mozaffarian D. (2016). Dietary and Policy Priorities for Cardiovascular Disease, Diabetes, and Obesity. Circulation.

[7] Cardoso S.M., Pereira O.R., Seca A.M.L., Pinto D.C.G.A., Silva A.M.S. (2015). Seaweeds as Preventive Agents for Cardiovascular Diseases: From Nutrients to Functional Foods. Mar. Drugs.

[8] Camargo-Ramos C.M., Correa-Bautista J.E., Correa-Rodríguez M., Ramírez-Vélez R. (2017). Dietary Inflammatory Index and Cardiometabolic Risk Parameters in Overweight and Sedentary Subjects. Int. J. Environ. Res. Public Health.

[9] Langcake P., Pryce R.J. (1976). The Production of Resveratrol by *Vitis Vinifera* and Other Members of the Vitaceae as a Response to Infection or Injury. Physiol. Plant Pathol..

[10] Burns J., Yokota T., Ashihara H., Lean M.E.J., Crozier A. (2002). Plant Foods and Herbal Sources of Resveratrol. J. Agric. Food Chem..

[11] European Commission (2018). COMMISSION IMPLEMENTING REGULATION (EU) 2021/51 of 22 January 2021 Authorising a Change of the Conditions of Use of the Novel Food ‘*Trans*-Resveratrol’ under Regulation (EU) 2015/2283 of the European Parliament and of the Council and Amending Commission Im. Off. J. Eur. Union.

[12] Borriello A., Cucciolla V., Della Ragione F., Galletti P. (2010). Dietary Polyphenols: Focus on Resveratrol, a Promising Agent in the Prevention of Cardiovascular Diseases and Control of Glucose Homeostasis. Nutr. Metab. Cardiovasc. Dis..

[13] Szkudelski T., Szkudelska K. (2011). Anti-Diabetic Effects of Resveratrol. Ann. N. Y. Acad. Sci..

[14] Sulaiman M., Matta M.J., Sunderesan N.R., Gupta M.P., Periasamy M., Gupta M. (2010). Resveratrol, an Activator of SIRT1, Upregulates Sarcoplasmic Calcium ATPase and Improves Cardiac Function in Diabetic Cardiomyopathy. Am. J. Physiol. Circ. Physiol..

[15] Szkudelska K., Deniziak M., Hertig I., Wojciechowicz T., Tyczewska M., Jaroszewska M., Szkudelski T. (2019). Effects of Resveratrol in Goto-Kakizaki Rat, a Model of Type 2 Diabetes. Nutrients.

[16] Huang J.-P., Huang S.-S., Deng J.-Y., Chang C.-C., Day Y.-J., Hung L.-M. (2010). Insulin and Resveratrol Act Synergistically, Preventing Cardiac Dysfunction in Diabetes, but the Advantage of Resveratrol in Diabetics with Acute Heart Attack Is Antagonized by Insulin. Free Radic. Biol. Med..

[17] Crandall J.P., Oram V., Trandafirescu G., Reid M., Kishore P., Hawkins M., Cohen H.W., Barzilai N. (2012). Pilot Study of Resveratrol in Older Adults With Impaired Glucose Tolerance. J. Gerontol. Ser. A.

[18] Cai H., Scott E., Kholghi A., Andreadi C., Rufini A., Karmokar A., Britton R.G., Horner-Glister E., Greaves P., Jawad D. (2015). Cancer Chemoprevention: Evidence of a Nonlinear Dose Response for the Protective Effects of Resveratrol in Humans and Mice. Sci. Transl. Med..

[19] Berman A.Y., Motechin R.A., Wiesenfeld M.Y., Holz M.K. (2017). The Therapeutic Potential of Resveratrol: A Review of Clinical Trials. NPJ Precis. Oncol..

[20] Hasan M.M., Yun H.-K., Kwak E.-J., Baek K.-H. (2014). Preparation of Resveratrol-Enriched Grape Juice from Ultrasonication Treated Grape Fruits. Ultrason. Sonochem..

[21] Gaudette N.J., Pickering G.J. (2011). Sensory and Chemical Characteristics of *Trans*-Resveratrol-Fortified Wine. Aust. J. Grape Wine Res..

[22] Silva A.F.R., Monteiro M., Resende D., Braga S.S., Coimbra M.A., Silva A.M.S., Cardoso S.M. (2020). Inclusion Complex of Resveratrol with γ-Cyclodextrin as a Functional Ingredient for Lemon Juices. Foods.

[23] Silva A.F.R., Monteiro M., Nunes R., Baião A., Braga S.S., Sarmento B., Coimbra M.A., Silva A.M.S., Cardoso S.M. (2022). Bread Enriched with Resveratrol: Influence of the Delivery Vehicles on Its Bioactivity. Food Biosci..

[24] Masood S., Rehman A.U., Bashir S., El Shazly M., Imran M., Khalil P., Ifthikar F., Jaffar H.M., Khursheed T. (2021). Investigation of the Anti-Hyperglycemic and Antioxidant Effects of Wheat Bread Supplemented with Onion Peel Extract and Onion Powder in Diabetic Rats. J. Diabetes Metab. Disord..

[25] Weng Y., Zhang M., Wang J., Zhang Y. (2021). Significantly Hypoglycemic Effect of a Novel Functional Bread Rich in Mulberry Bark and Improving the Related Functions of Liver, Pancreas, and Kidney, on T2D Mice. Food Sci. Nutr..

[26] Tan Y., Zhang Z., Zheng C., Wintergerst K.A., Keller B.B., Cai L. (2020). Mechanisms of Diabetic Cardiomyopathy and Potential Therapeutic Strategies: Preclinical and Clinical Evidence. Nat. Rev. Cardiol..

[27] Wilson R.D., Islam M.S. (2012). Fructose-Fed Streptozotocin-Injected Rat: An Alternative Model for Type 2 Diabetes. Pharmacol. Rep..

[28] Adoga J.O., Channa M.L., Nadar A. (2021). Kolaviron Attenuates Cardiovascular Injury in Fructose-Streptozotocin Induced Type-2 Diabetic Male Rats by Reducing Oxidative Stress, Inflammation, and Improving Cardiovascular Risk Markers. Biomed. Pharmacother..

[29] Castelhano J., Ribeiro B., Sanches M., Graça B., Saraiva J., Oliveiros B., Neves C., Rodrigues T., Sereno J., Gonçalves S. (2020). A Rat Model of Enhanced Glycation Mimics Cardiac Phenotypic Components of Human Type 2 Diabetes: A Translational Study Using MRI. J. Diabetes Complicat..

[30] Silva-Reis R., Faustino-Rocha A.I., Silva J., Valada A., Azevedo T., Anjos L., Gonçalves L., Pinto M.d.L., Ferreira R., Silva A.M.S. (2023). Studying and Analyzing Humane Endpoints in the Fructose-Fed and Streptozotocin-Injected Rat Model of Diabetes. Animals.

[31] Olfert E., Bhasin J., Latt R., Macallum E., McCutcheon K., Rainnie D., Schunk M. (1998). CCAC Guidelines on: Choosing an Appropriate Endpoint in Experiments Using Animals for Research, Teaching and Testing.

[32] Cacho J., Sevillano J., De Castro J., Herrera E., Ramos M.P. (2008). Validation of Simple Indexes to Assess Insulin Sensitivity during Pregnancy in Wistar and Sprague-Dawley Rats. Am. J. Physiol.-Endocrinol. Metab..

[33] Brandão S.R., Reis-Mendes A., Domingues P., Duarte J.A., Bastos M.L., Carvalho F., Ferreira R., Costa V.M. (2021). Exploring the Aging Effect of the Anticancer Drugs Doxorubicin and Mitoxantrone on Cardiac Mitochondrial Proteome Using a Murine Model. Toxicology.

[34] Padrão A.I., Carvalho T., Vitorino R., Alves R.M.P., Caseiro A., Duarte J.A., Ferreira R., Amado F. (2012). Impaired Protein Quality Control System Underlies Mitochondrial Dysfunction in Skeletal Muscle of Streptozotocin-Induced Diabetic Rats. Biochim. Biophys. Acta-Mol. Basis Dis..

[35] Silva M.G., Nunes P., Oliveira P., Ferreira R., Fardilha M., Moreira-Gonçalves D., Duarte J.A., Oliveira M.M., Peixoto F. (2022). Long-Term Aerobic Training Improves Mitochondrial and Antioxidant Function in the Liver of Wistar Rats Preventing Hepatic Age-Related Function Decline. Biology.

[36] Fajardo R.J., Karim L., Calley V.I., Bouxsein M.L. (2014). A Review of Rodent Models of Type 2 Diabetic Skeletal Fragility. J. Bone Miner. Res..

[37] Barrière D.A., Noll C., Roussy G., Lizotte F., Kessai A., Kirby K., Belleville K., Beaudet N., Longpré J.M., Carpentier A.C. (2018). Combination of High-Fat/High-Fructose Diet and Low-Dose Streptozotocin to Model Long-Term Type-2 Diabetes Complications. Sci. Rep..

[38] He Q., Su G., Liu K., Zhang F., Jiang Y., Gao J., Liu L., Jiang Z., Jin M., Xie H. (2017). Sex-Specific Reference Intervals of Hematologic and Biochemical Analytes in Sprague-Dawley Rats Using the Nonparametric Rank Percentile Method. PLoS ONE.

[39] Maintra A. (2015). The Endocrine System. Robbins & Cotran–Pathologic Basis of Disease.

[40] Jeyaraman M.M., Srisakuldee W., Nickel B.E., Kardami E. (2012). Connexin43 Phosphorylation and Cytoprotection in the Heart. Biochim. Biophys. Acta-Biomembr..

[41] Boström P., Mann N., Wu J., Quintero P.A., Plovie E.R., Panáková D., Gupta R.K., Xiao C., MacRae C.A., Rosenzweig A. (2010). C/EBPβ Controls Exercise-Induced Cardiac Growth and Protects against Pathological Cardiac Remodeling. Cell.

[42] Huang G.N., Thatcher J.E., McAnally J., Kong Y., Qi X., Tan W., DiMaio J.M., Amatruda J.F., Gerard R.D., Hill J.A. (2012). C/EBP Transcription Factors Mediate Epicardial Activation During Heart Development and Injury. Science.

[43] Ding S., Gan T., Song M., Dai Q., Huang H., Xiao J. (2017). C/EBPB-CITED4 in Exercised Heart. Exercise for Cardiovascular Disease Prevention and Treatment.

[44] Aerni-Flessner L., Abi-Jaoude M., Koenig A., Payne M., Hruz P.W. (2012). GLUT4, GLUT1, and GLUT8 Are the Dominant GLUT Transcripts Expressed in the Murine Left Ventricle. Cardiovasc. Diabetol..

[45] Houten S.M., Wanders R.J.A. (2010). A General Introduction to the Biochemistry of Mitochondrial Fatty Acid Β-oxidation. J. Inherit. Metab. Dis..

[46] Sun W., Liu C., Chen Q., Liu N., Yan Y., Liu B. (2018). SIRT3: A New Regulator of Cardiovascular Diseases. Oxid. Med. Cell. Longev..

[47] Castro L., Demicheli V., Tórtora V., Radi R. (2011). Mitochondrial Protein Tyrosine Nitration. Free Radic. Res..

[48] Chakarova N., Dimova R., Grozeva G., Tankova T. (2019). Assessment of Glucose Variability in Subjects with Prediabetes. Diabetes Res. Clin. Pract..

[49] Palsamy P., Subramanian S. (2008). Resveratrol, a Natural Phytoalexin, Normalizes Hyperglycemia in Streptozotocin-Nicotinamide Induced Experimental Diabetic Rats. Biomed. Pharmacother..

[50] Baynest H.W. (2015). Classification, Pathophysiology, Diagnosis and Management of Diabetes Mellitus. J. Diabetes Metab..

[51] Zhang F., Ye C., Li G., Ding W., Zhou W., Zhu H., Chen G., Luo T., Guang M., Liu Y. (2003). The Rat Model of Type 2 Diabetic Mellitus and Its Glycometabolism Characters. Exp. Anim..

[52] Palsamy P., Subramanian S. (2010). Ameliorative Potential of Resveratrol on Proinflammatory Cytokines, Hyperglycemia Mediated Oxidative Stress, and Pancreatic β-Cell Dysfunction in Streptozotocin-Nicotinamide-Induced Diabetic Rats. J. Cell. Physiol..

[53] Burgeiro A., Cerqueira M., Varela-Rodríguez B., Nunes S., Neto P., Pereira F., Reis F., Carvalho E. (2017). Glucose and Lipid Dysmetabolism in a Rat Model of Prediabetes Induced by a High-Sucrose Diet. Nutrients.

[54] Han L., Bittner S., Dong D., Cortez Y., Bittner A., Chan J., Umar M., Shen W.-J., Peterson R.G., Kraemer F.B. (2020). Molecular Changes in Hepatic Metabolism in ZDSD Rats–A New Polygenic Rodent Model of Obesity, Metabolic Syndrome, and Diabetes. Biochim. Biophys. Acta-Mol. Basis Dis..

[55] Seedevi P., Ramu Ganesan A., Moovendhan M., Mohan K., Sivasankar P., Loganathan S., Vairamani S., Shanmugam A. (2020). Anti-Diabetic Activity of Crude Polysaccharide and Rhamnose-Enriched Polysaccharide from G. Lithophila on Streptozotocin (STZ)-Induced in Wistar Rats. Sci. Rep..

[56] Mohan M., Dihoum A., Mordi I.R., Choy A.-M., Rena G., Lang C.C. (2021). Left Ventricular Hypertrophy in Diabetic Cardiomyopathy: A Target for Intervention. Front. Cardiovasc. Med..

[57] Rospleszcz S., Schafnitzel A., Koenig W., Lorbeer R., Auweter S., Huth C., Rathmann W., Heier M., Linkohr B., Meisinger C. (2018). Association of Glycemic Status and Segmental Left Ventricular Wall Thickness in Subjects without Prior Cardiovascular Disease: A Cross-Sectional Study. BMC Cardiovasc. Disord..

[58] Eguchi K., Boden-Albala B., Jin Z., Rundek T., Sacco R.L., Homma S., Di Tullio M.R. (2008). Association Between Diabetes Mellitus and Left Ventricular Hypertrophy in a Multiethnic Population. Am. J. Cardiol..

[59] Brands M.W., Fitzgerald S.M., Hewitt W.H., Hailman A.E. (2000). Decreased Cardiac Output at the Onset of Diabetes: Renal Mechanisms and Peripheral Vasoconstriction. Am. J. Physiol. Endocrinol. Metab..

[60] Chen X.-F., Wang J.-A., Lin X.-F., Tang L.-J., Yu W.-F., Chen H., Xie X.-J., Jiang J.-J., Peng X.-H. (2009). Diabetes Mellitus: Is It Protective against Aortic Root Dilatation?. Cardiology.

[61] Ehl N.F., Kühne M., Brinkert M., Müller-Brand J., Zellweger M.J. (2011). Diabetes Reduces Left Ventricular Ejection Fraction--Irrespective of Presence and Extent of Coronary Artery Disease. Eur. J. Endocrinol..

[62] Akula A. (2003). Biochemical, Histological and Echocardiographic Changes during Experimental Cardiomyopathy in STZ-Induced Diabetic Rats. Pharmacol. Res..

[63] Gordan R., Gwathmey J.K., Xie L. (2015). Autonomic and Endocrine Control of Cardiovascular Function. World J. Cardiol..

[64] Hensel K.O. (2016). Non-Ischemic Diabetic Cardiomyopathy May Initially Exhibit a Transient Subclinical Phase of Hyperdynamic Myocardial Performance. Med. Hypotheses.

[65] Patil M.B., Burji N.P.A. (2012). Echocardiographic Evaluation of Diastolic Dysfunction in Asymptomatic Type 2 Diabetes Mellitus. J. Assoc. Physicians India.

[66] Jia G., Whaley-Connell A., Sowers J.R. (2018). Diabetic Cardiomyopathy: A Hyperglycaemia- and Insulin-Resistance-Induced Heart Disease. Diabetologia.

[67] Diao J., Wei J., Yan R., Fan G., Lin L., Chen M. (2019). Effects of Resveratrol on Regulation on UCP2 and Cardiac Function in Diabetic Rats. J. Physiol. Biochem..

[68] Kanamori H., Takemura G., Goto K., Tsujimoto A., Mikami A., Ogino A., Watanabe T., Morishita K., Okada H., Kawasaki M. (2015). Autophagic Adaptations in Diabetic Cardiomyopathy Differ between Type 1 and Type 2 Diabetes. Autophagy.

[69] Wu H., Li G.N., Xie J., Li R., Chen Q.H., Chen J.Z., Wei Z.H., Kang L.N., Xu B. (2016). Resveratrol Ameliorates Myocardial Fibrosis by Inhibiting ROS/ERK/TGF-β/Periostin Pathway in STZ-Induced Diabetic Mice. BMC Cardiovasc. Disord..

[70] Sakamoto T., Kelly D.P. (2020). A Case for Adaptive Cardiac Hypertrophic Remodeling Is CITED. Circ. Res..

[71] Radosinka J., Kurahara L.H., Hirashi K., Viczencozva C., Egan T.B., Szeiffova B.B., Dosenko V., Navarova J., Obsitnik B., Imanaga I. (2015). Modulation of Cardiac Connexin-43 by Omega-3 Fatty Acid Ethyl-Ester Supplementation Demonstrated in Spontaneously Diabetic Rats. Physiol. Res..

[72] Joshi M.S., Mihm M.J., Cook A.C., Schanbacher B.L., Bauer J.A. (2015). Alterations in Connexin 43 during Diabetic Cardiomyopathy: Competition of Tyrosine Nitration versus Phosphorylation. J. Diabetes.

[73] Karwi Q.G., Uddin G.M., Ho K.L., Lopaschuk G.D. (2018). Loss of Metabolic Flexibility in the Failing Heart. Front. Cardiovasc. Med..

[74] Mazumder P.K., O’Neill B.T., Roberts M.W., Buchanan J., Yun U.J., Cooksey R.C., Boudina S., Abel E.D. (2004). Impaired Cardiac Efficiency and Increased Fatty Acid Oxidation in Insulin-Resistant Ob/Ob Mouse Hearts. Diabetes.

[75] Stanley W.C., Chandler M.P. (2002). Energy Metabolism in the Normal and Failing Heart: Potential for Therapeutic Interventions. Heart Fail. Rev..

[76] Verma S.K., Garikipati V.N.S., Kishore R. (2017). Mitochondrial Dysfunction and Its Impact on Diabetic Heart. Biochim. Biophys. Acta–Mol. Basis Dis..

[77] Fadini G.P., Miorin M., Facco M., Bonamico S., Baesso I., Grego F., Menegolo M., de Kreutzenberg S.V., Tiengo A., Agostini C. (2005). Circulating Endothelial Progenitor Cells Are Reduced in Peripheral Vascular Complications of Type 2 Diabetes Mellitus. J. Am. Coll. Cardiol..

[78] Bagul P., Katare P., Bugga P., Dinda A., Banerjee S.K. (2018). SIRT-3 Modulation by Resveratrol Improves Mitochondrial Oxidative Phosphorylation in Diabetic Heart through Deacetylation of TFAM. Cells.

[79] Chen T., Li J., Liu J., Li N., Wang S., Liu H., Zeng M., Zhang Y., Bu P. (2015). Activation of SIRT3 by Resveratrol Ameliorates Cardiac Fibrosis and Improves Cardiac Function via the TGF-β/Smad3 Pathway. Am. J. Physiol. Circ. Physiol..

[80] Gumede N., Ngubane P., Khathi A. (2022). Assessing the Risk Factors for Myocardial Infarction in Diet-Induced Prediabetes: Myocardial Tissue Changes. BMC Cardiovasc. Disord..

