# Supplementary material for: The Impact of Resveratrol-Enriched Bread on Cardiac Remodeling in a Preclinical Model of Diabetes
Source: Antioxidants (Basel). 2023 May 9;12(5):1066. doi: 10.3390/antiox12051066 (PMC10215477; doi:10.3390/antiox12051066)
Supplement: Supplementary file 1 [file antioxidants-12-01066-s001.zip › antioxidants-2330180-supplementary.pdf]

Supplementary data

The Impact of Resveratrol-Enriched Bread on Cardiac Remodeling in a Preclinical Model of Diabetes

1. Experimental groups identification

Forty-eight outbred wild male Sprague-Dawley rats with three weeks old were randomly divided into four groups, represented in the Table S1, and stayed one week for acclimatization with standard diet and further fed with bread diet up to the third week of the experiment (Figure S1).

Table S1. Animals group and respective treatment of experimental protocol.

Treatment	Group name
Control + Bread	CB
Control + Bread + RSV (10 mg/Kg)	CBR
Diabetes + Bread	DB
Diabetes + Bread + RSV (10 mg/Kg)	DBR

2. **Animals' groups monitorization on anthropometric and welfare parameters along experiment**

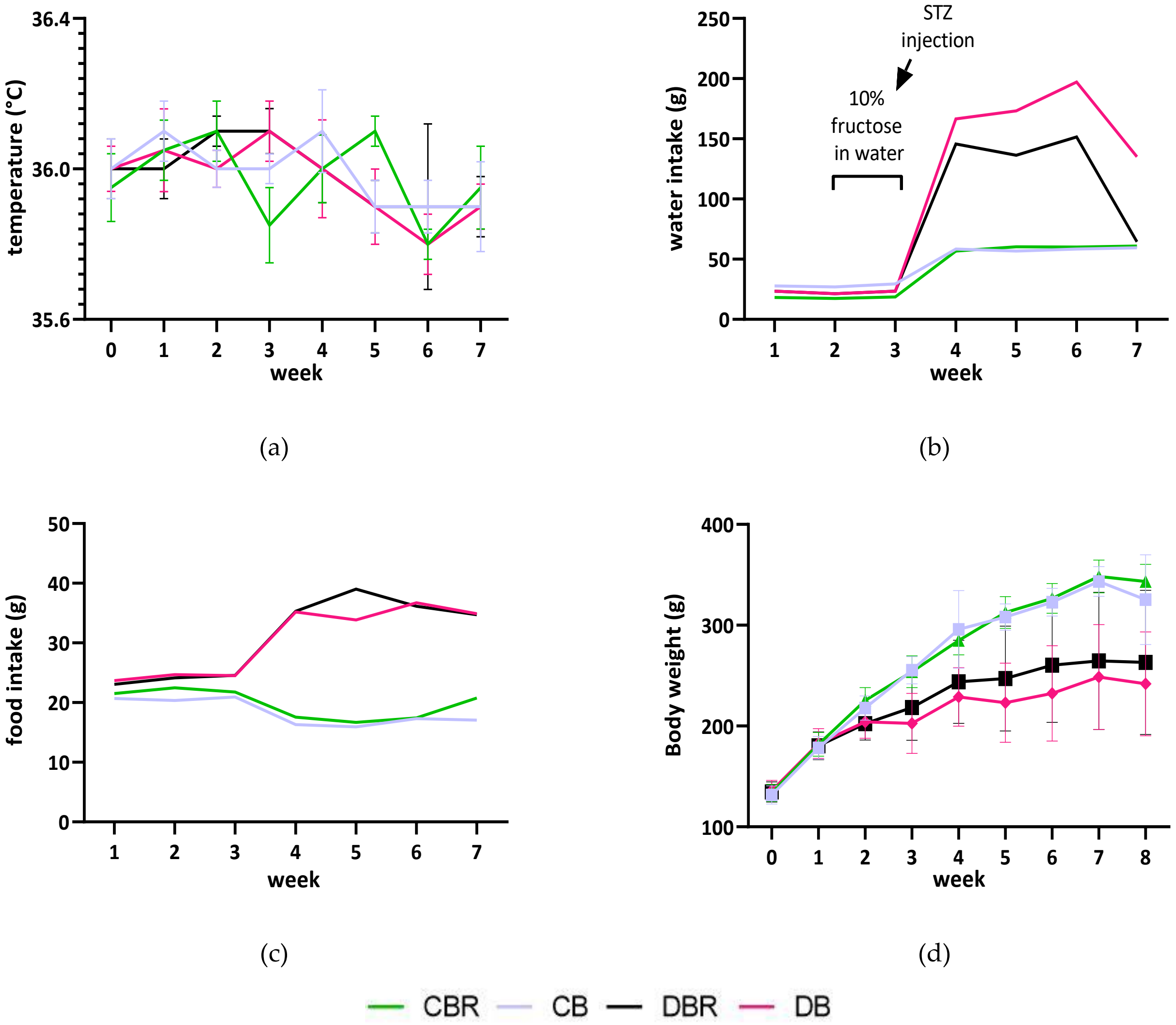

Figure S1. Weekly parameters of animal's housing conditions: temperature (a), water (b) and food intake (c) and body weight (d). Body weight at week 8 corresponds to the day of the necropsy. CB is the control group fed with plain bread (n = 8) , and CBR is the group fed bread with 10 mg/kg of RSV a day (n = 8); DB is the fructose/STZ group fed with plain bread (n = 15), and DBR is the one fed with bread containing 10 mg/kg of RSV a day (n = 16).

Table S2. Humane Endpoints applied to evaluate animal welfare in a fructose/STZ-induced diabetes rat model.

Score		0	1	2	3
Body Condition / Weight		Normal	Altered body condition / weight loss of <10%	Emaciated / weight loss of 10-20%	Weight loss of > 20% (Euthanasia)
Posture		Normal	Curved	---	---
Hair/tail appearance and grooming		Normal	Lack of grooming	Very bad looking hair, dirty tail, severe chromodachryorrhea	
Grimace scale	Eyes and extremities	Normal	Narrowing of the orbital area	Moderate anemia/Cataracts, Eyes fully closed	Severe anemia/Corneal ulcers (Euthanasia)
	Position of ears and whiskers	Normal	Dropped ears, forward whiskers (Stiffened)	Floppy and curved ears, whiskers turned	---

			forward and crumpled (clusters)	
Nose/cheeks	Normal	Flattening and elongating the tip of the nose	Flattening of the cheeks (cheek appearance)	---
Walk	Normal	---	---	Walking on the tip of the extremities (Euthanasia)
Skin	Absence	Skin injuries/infections < 5mm	Skin injuries/infections > 5mm	Presence of necrosis (Euthanasia)
Mental status	Normal	Inactive	Moribund	Stupor/coma (Euthanasia)
Response to external stimuli	Normal	Moderate	Moderate with vocalization	Violent
Hydration status	Normal	Abnormal skin pinch test (> 2 sec)	---	---
Stool appearance	Solid	Pasty diarrhea	Liquid diarrhea	---
Convulsions	Absence	---	---	Presence
Visualization and response to mild abdominal palpation	Normal	Vocalization	Distended	---

Recommendation: If an animal's total score was 4 or higher or a score of 3 in some parameters, such as body weight, it was re-evaluated to decide if it should be removed from the protocol.

3. Glycaemia monitorization along animal experiment

Glucose measures were taken at week 4 of experiment, before the introduction of the diet with bread and two and four weeks later to evaluate diabetes development (Figure S2.a-b). The feeding glucose levels of both control groups with values lower than 200 mg/ dL was due to its efficient response to keep glucose homeostasis. Fasting glucose levels were lower than 200 mg/ dL in fructose/STZ animals.

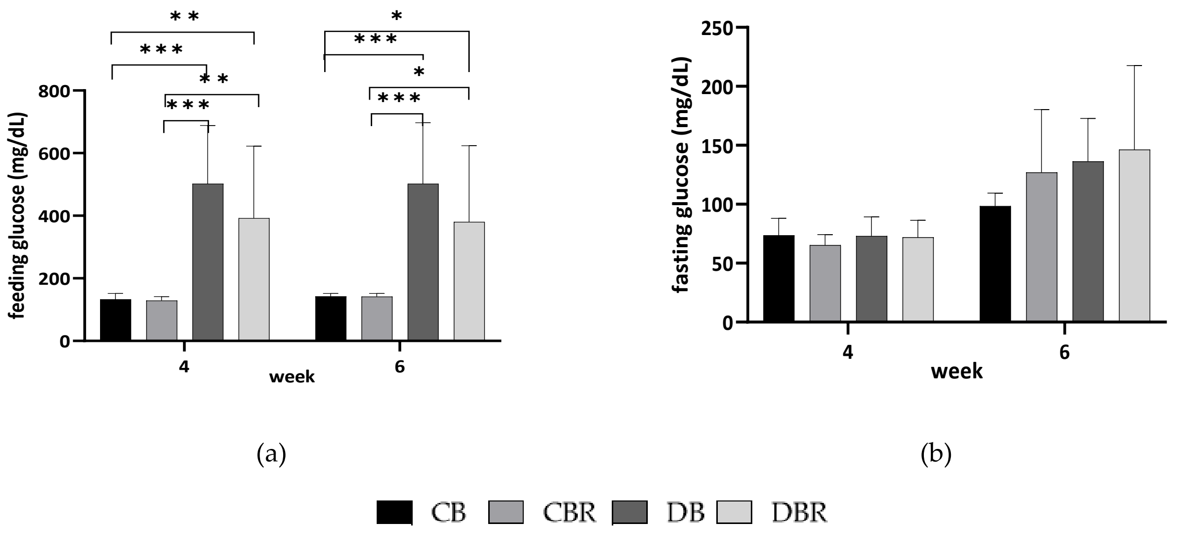

Figure S2. Circulating glucose levels after feeding (a) and fasting (b) moments of Sprague-Dawley rats in the final of the experiment. Values represent mean ± standard deviation (* $p < 0.05$, ** $p < 0.01$ and *** $p < 0.001$). CB is the control group fed with plain bread (n = 8), and CBR is the group fed bread with 10 mg/kg of RSV a day (n = 8); DB is the fructose/STZ group fed with plain bread (n = 15), and DBR is the one fed with bread containing 10 mg/kg of RSV a day (n = 16).

4. Echocardiogram parameters for evaluating cardiac function

Table S3. All *e*chocardiogram parameters of animal experiment.

	Groups of Treatment			
	CB	CBR	DB	DBR
BSA (cm^2)	429.6± 41.2	446.0± 14.8	328.2 ± 22.6	359.6 ± 77.9
IVS_d (mm)	1.12 ± 0.18	1.21 ± 0.20	0.97 ± 0.17	1.40 ± 0.26
LV_d	7.28 ± 0.99	7.46 ± 0.74	7.73 ± 1.05	7.01 ± 1.39
$LVPW_d$ (mm)	1.11 ± 0.18	1.29 ± 0.29	0.99 ± 0.16	1.51 ± 0.34
IVS_s (mm)	1.17 ± 0.23	1.17 ± 0.18	0.96 ± 0.17	1.47 ± 0.24
LV_s (mm)	6.84 ± 1.20	6.55 ± 0.50	7.07 ± 1.08	6.01 ± 1.44
$LVPW_s$ (mm)	1.21 ± 0.19	1.21 ± 0.24	0.93 ± 0.21	1.65 ± 0.38
Ao_d (cm)	0.28 ± 0.04	0.30 ± 0.03	0.31 ± 0.03	0.28 ± 0.05
Ao VTI (cm)	3.94 ± 0.86	5.23 ± 1.20	4.30 ± 0.82	3.81 ± 1.21
HR (bpm or min^{-1})	321.3 ± 25.8	272.5 ± 37.9	190.0 ± 36.7	217.5 ± 67.3
LV ET (s)	82.9 ± 8.37	93.9 ± 18.3	125.8 ± 18.5	108.9 ± 20.0
E (cm/s)	0.68 ± 0.12	0.81 ± 0.11	0.60 ± 0.19	0.58 ± 0.17
A (cm/s)	0.43 ± 0.10	0.38 ± 0.15	0.39 ± 0.12	0.31 ± 0.07
AE (cm^2)	13.6 ± 3.9	12.0 ± 2.8	10.1 ± 3.5	10.8 ± 3.3
LV mass (mm)	0.50 ± 0.10	0.60 ± 0.13	0.48 ± 0.12	0.73 ± 0.25
FS (%)	6.69 ± 3.30	15.4 ± 7.4	11.2 ± 4.76	13.4 ± 6.9
SV (mL)	1.12 ± 0.30	1.56 ± 0.37	1.36 ± 0.22	1.10 ± 0.45
CO (mL/min)	357.6 ± 98.1	420.6 ± 98.2	241.2 ± 39.9	251.7 ± 142.7
E/A	1.64 ± 0.29	2.47 ± 1.01	1.57 ± 0.21	1.90 ± 0.49
EF (%)	20.1 ± 10.1	38.3 ± 14.7	27.9 ± 11.3	37.6 ± 17.1
TAPSE (mm)	0.19 ± 0.04	0.26 ± 0.04	0.22 ± 0.05	0.16 ± 0.06
VTI AP (cm)	2.96 ± 0.84	3.67 ± 0.93	3.67 ± 2.19	3.72 ± 1.27
Pulmonary artery diameter (cm)	0.21 ± 0.04	0.21 ± 0.03	0.20 ± 0.03	0.19 ± 0.03
PAAT (ms)	31.3 ± 6.73	32.1 ± 7.2	29.5 ± 9.0	28.6 ± 4.09
SV	0.62 ± 0.22	0.78 ± 0.23	0.74 ± 0.51	0.73 ± 0.30
CO	195.7 ± 62.5	207.2 ± 51.0	137.1± 87.9	161.8 ± 89.9
AD (cm^2)	14.1 ± 4.1	12.4 ± 2.9	10.2 ± 1.8	10.6 ± 2.9
D1	5.50 ± 0.58	5.80 ± 0.65	5.66 ± 1.11	5.74 ± 0.98
D2	5.78 ± 0.55	5.29 ± 0.55	4.78 ± 0.89	5.21 ± 0.84
Eccentricity index (D1/D2)	0.96 ± 0.15	1.07 ± 0.14	1.20 ± 0.22	1.10 ± 0.12

Values are means ± standard deviation of n animals in each group. In each line, d in subscript means diastole and s in subscript means systole; BSA - body surface area; IVS - intraventricular septum thickness; LV - left ventricle; LVPW - left ventricular posterior wall thickness; Ao - aorta; Ao VTI - aortic velocity time integral; HR - heart rate; LV ET - left ventricular ejection time; E - peak early diastolic transmitral flow velocity; A - peak late transmitral flow velocity; AE – area of left atrium; FS - fractional shortening; SV - stroke volume; CO - cardiac output; EF - ejection fraction; TAPSE -

tricuspid annular plane systolic excursion; VTI AP - pulmonary velocity time integral; PAAT - pulmonary artery acceleration time; SV – stroke volume; CO – cardiac output; AD - area of right atrium; D1 and D2 - length of two perpendicular minor-axis of the left ventricle. CB is the control group fed with plain bread (n = 8), and CBR is the group fed bread with 10 mg/kg of RSV a day (n = 8); DB is the fructose/STZ group fed with plain bread (n = 7), and DBR is the one fed with bread containing 10 mg/kg of RSV a day (n = 8).

5. Antibodies information

Table S4. Specification of the dilutions and catalog number for each primary antibody.

Primary antibody	Catalog number	Dilution used	Molecular weight of targer protein (kDa)	Western blot	Slot blot
CRP	ab65842	1:1000	-		X
ATPβ	ab14730	1:1000	52	X	
PFKM	ab154804	1:1000	85	X	
CEBPβ	ab32358	1:1000	36	X	
CITED4	mbs833529	1:1000	17	X	
Cx43	ab11370	1:1000	43	X	
SIRT3	26275	1:1000	28	X	
GLUT4	ab48547	1:1000	30	X	
MnSOD	ab13533	1:1000	25	X	
ETFDH	ab91505	1:1000	69	X	
DNP	MAB2223	1:1000	-	X	X
nitroTyr	MAB5404	1:1000	-		X
